# Repertory of Tongue Symptoms

**Published:** 1896-11

**Authors:** 


Repertory of Tongue Symptoms. Arranged by M. E. Douglass, M. D., Baltimore, Md. Philadelphia: Bœricke & Tafel, 1896. Price, in cloth, $1.00; by mail, $1.09.

This valuable little monograph of 190 pages is a welcome addition to the library of repertories that every homœopathic physician must have who undertakes to prescribe strictly according to Hahnemann's system. This little repertory is arranged strictly on the alphabetical plan. The principal words are printed in heavy black type the better to catch the eye, and the sentences which depend upon that principal word are in ordinary small primer together with the names of the medicines.

In some of the indications it is a trifle deficient. Thus in the rubric "Imprint of the teeth on the tongue," the author has failed to include the following remedies: Arsenicum-metallicum, Antimonium-tartaricum, Ignatia, Iodine, Glonoine, Tellurium, and Kali-bichromicum. This last remedy is, however, given under the rubric "Scalloped."

Under the rubric "Scalded," however, the various indications are very satisfactory and the list of remedies given is quite full. All who buy the book should from time to time add notes where there are omissions discovered, and these should be forwarded to the author so that he may get out an improved second edition, for we have no doubt that a second edition will be needed. No repertory of the tongue has, as far as we know, ever been issued in a separate volume, and we have no doubt the profession will welcome this little book.

